# Diagnostic Assessment of Endoscopic Ultrasonography–Fine Needle Aspiration Cytology in the Pancreas: A Comparison between Liquid-Based Preparation and Conventional Smear

**DOI:** 10.3390/medicina60060930

**Published:** 2024-06-02

**Authors:** Jung-Soo Pyo, Dae Hyun Lim, Kyueng-Whan Min, Nae Yu Kim, Il Hwan Oh, Byoung Kwan Son

**Affiliations:** 1 Department of Pathology, Uijeongbu Eulji Medical Center, Eulji University School of Medicine, Uijeongbu-si 11759, Republic of Korea; jspyo@eulji.ac.kr (J.-S.P.); kyueng@eulji.ac.kr (K.-W.M.); 2 Department of Internal Medicine, Uijeongbu Eulji Medical Center, Eulji University School of Medicine, Uijeongbu-si 11759, Republic of Korea; daehyun.lim@eulji.ac.kr (D.H.L.); naeyu46@eulji.ac.kr (N.Y.K.); 20180121@eulji.ac.kr (I.H.O.)

**Keywords:** pancreas, endoscopic ultrasonography, fine needle aspiration cytology, liquid-based preparation, conventional smear, decision tree analysis

## Abstract

*Background and Objectives*: This study aimed to elucidate the cytologic characteristics and diagnostic usefulness of endoscopic ultrasonography–fine needle aspiration cytology (EUS-FNAC) by comparing it with liquid-based preparation (LBP) and conventional smear (CS) in pancreas. *Methods*: The diagnostic categories (I through VII) were classified according to the World Health Organization Reporting System for Pancreaticobiliary Cytopathology. Ten cytologic features, including nuclear and additional features, were evaluated in 53 cases subjected to EUS-FNAC. Nuclear features comprised irregular nuclear contours, nuclear enlargement, hypochromatic nuclei with parachromatin clearing, and nucleoli. Additional cellular features included isolated atypical cells, mucinous cytoplasm, drunken honeycomb architecture, mitosis, necrotic background, and cellularity. A decision tree analysis was conducted to assess diagnostic efficacy. *Results*: The diagnostic concordance rate between LBP and CS was 49.1% (26 out of 53 cases). No significant differences in nuclear features were observed between categories III (atypical), VI (suspicious for malignancy), and VII (malignant). The decision tree analysis of LBP indicated that cases with moderate or high cellularity and mitosis could be considered diagnostic for those exhibiting nuclear atypia. Furthermore, in CS, mitosis, isolated atypical cells, and necrotic background exerted a more significant impact on the diagnosis of EUS-FNAC. *Conclusions*: Significant parameters for interpreting EUS-FNAC may differ between LBP and CS. While nuclear atypia did not influence the diagnosis of categories III, VI, and VII, other cytopathologic features, such as cellularity, mitosis, and necrotic background, may present challenges in diagnosing EUS-FNAC.

## 1. Introduction

Pancreatic ductal adenocarcinoma constitutes the majority of malignant pancreatic tumors in adults [1]. Endoscopic ultrasonography–fine needle aspiration cytology (EUS-FNAC) was introduced in the 1990s and has proven increasingly pivotal for diagnosing pancreatic lesions [2,3]. Due to the challenging anatomical location of the pancreas, acquiring a sufficient sample for diagnosis may be inherently challenging. The cytopathologic findings in the obtained EUS-FNAC specimen may carry significant diagnostic weight. In daily practice, to improve sample adequacy, the application of rapid on-site evaluation (ROSE) can increase sample adequacy [3]. Liquid-based preparation (LBP) stands as a viable method for preparing and evaluating EUS-FNAC specimens of pancreatic lesions. Comparative studies between conventional smears (CS) and LBP for EUS-FNAC have been documented [3]. Despite the potential complications associated with EUS-FNA, the method proves effective in evaluating pancreatic lesions [4]. The adequacy of samples obtained through EUS-FNA ranges from 82% to 91%, while the diagnostic sensitivity for malignant tumors spans from 64% to 96% [5,6,7,8,9,10,11,12,13,14,15,16,17,18]. Several factors can influence sample adequacy and diagnostic accuracy. The impact of cytopathologic findings on diagnostic accuracy has received comparatively less attention in the literature.

Two primary systems for reporting EUS-FNAC results are the Papanicolaou Society of Cytopathology (PSC) and the World Health Organization (WHO) reporting systems [1,19]. Recent studies have focused on comparing the relevance of these two reporting systems [20,21]. The use of a reporting system is expected to contribute to achieving diagnostic consistency and enhancing communication. Additionally, employing a reporting system is expected to reduce the frequency of atypical interpretations. In the WHO reporting system, the key diagnostic features of ductal adenocarcinoma are described as follows [1]: (1) usually highly cellular smears with mostly tissue fragments; (2) loosely cohesive or crowded tissue fragments with or without isolated tumor cells; (3) irregular nuclear spacing and a loss of polarity in sheets (drunken honeycomb architecture); (4) clean background in well-differentiated pancreatic ductal adenocarcinoma (PDAC), varying to abundant necrosis in high-grade PDAC; (5) mucinous cytoplasm, sometimes finely vacuolated and lacy, resulting in a deceptively low N:C ratio; (6) hypochromatic nuclei with parachromatin clearing, varying to hyperchromatic nuclei with irregular nuclear contours; (7) nucleoli may be present and prominent; (8) atypical mitotic figures; and (9) background necrosis often present, but not always. In the atypical category, the key diagnostic features are described as follows: (1) loss of architectural polarity; (2) minor degree of nuclear crowding; (3) mild alteration in the honeycomb pattern; (4) no 3D tissue fragments; (5) no true nuclear molding; (6) pseudostratification within the lower two-thirds of cell strips; (7) near-normal N–C ratio; (8) slight nuclear membrane irregularity without marked clefting; (9) parachromatic clearing without other cytopathological features of adenocarcinoma; (10) small nucleoli without true macronucleoli; (11) minor anisonucleosis, 2:1; and (12) clean background without necrosis. An atypical category diagnosis may be assigned if the cytologic features are insufficient to definitively determine malignancy or benignity. In cases exhibiting suspected malignant cytologic features, a diagnosis may be categorized as atypical due to low cellularity. Reducing the number of atypical category diagnoses requiring repeat studies is valuable in daily practice. However, existing literature has primarily focused on the implications of the atypical category for rates of malignancy (ROMs). Further studies are required to explore the diagnostic impact of each nuclear and cellular feature [22]. Within pancreaticobiliary cytology, the importance of ancillary tests has grown. To conduct ancillary tests, obtaining LBP, or biopsy samples, is imperative. Employing LBP alone for both diagnostic and ancillary tests, excluding CS, may offer advantages. This study aimed to evaluate cytopathologic features based on the diagnostic categories and methods used in the EUS-FNAC of pancreas. Our investigation focused on cytopathologic features based on the WHO reporting system. We evaluated the concordances in cellularity and diagnostic category between CS and LBP. Additionally, the diagnostic implications of cytopathologic features were assessed through a decision tree analysis.

## 2. Materials and Methods

### 2.1. Patients

We evaluated the cytopathological characteristics of 53 cases of EUS-FNAC suspected malignancy at the Department of Pathology, Uijeonbu Eulji Medical Center, Eulji University School of Medicine (Republic of Korea), from 1 April 2021 to 30 June 2023. For diagnosis, all cytological slides of cases were produced using two methods: LBP and CS. On EUS-FNAC, 53 cases were diagnosed as adenocarcinoma (n = 16), suspected adenocarcinoma (n = 12), or atypical (n = 25). Two pathologists reviewed the cytological slides. Cytopathological features were evaluated using the WHO Reporting System for Pancreaticobiliary Cytopathology. The Institutional Review Board (IRB) of Uijeongbu Eulji University Hospital reviewed and approved the study protocol. This study was exempt from obtaining informed consent through IRB review.

### 2.2. Evaluation of Cytological Features

Cytopathological features were evaluated by dividing them into nuclear and additional features. Nuclear features included irregular nuclear contours, nuclear enlargement, hypochromatic nuclei with parachromatin clearing, and nucleoli (Table 1). Other features included isolated atypical cells, mucinous cytoplasm, a drunken honeycomb architecture, mitosis, a necrotic background, and cellularity.

### 2.3. Statistical Analysis

Statistical analyses used SPSS version 22.0 software (SPSS, Chicago, IL, USA). The significance of the correlation between the number of cytopathological features and cytological diagnosis was determined using a two-tailed Student’s t-test. Results were considered statistically significant at *p* < 0.05.

Decision tree analysis was performed to investigate the diagnostic impact of cytopathological features on EUS-FNAC. A decision tree is represented by tree-like structures showing the nodes, leaves, and branches that classify the data. The nodes include root, parent, child, internal, and terminal nodes. The root node is the starting point that provides all the variables. The parent and child nodes are the upstream and downstream nodes, respectively, of a given node. A terminal node is considered as a node without a child node. An internal node has both parent and child nodes corresponding to each question. A branch labeled with an attribute is defined as a node that connects the root node to the terminal nodes. Consequently, the terminal nodes showed a decision or class corresponding to one of the diagnostic categories in our study. The decision tree was generated using an algorithm branching from the most important or basic variables through the following variables into diagnoses in sequential steps for clear differentiation of diagnostic categories. The decision tree analysis was performed using the rpart package in the R program [23].

## 3. Results

### 3.1. Cytopathologic Characteristics of Endoscopic Ultrasonography–Fine Needle Aspiration Cytology

**Figure 1:**
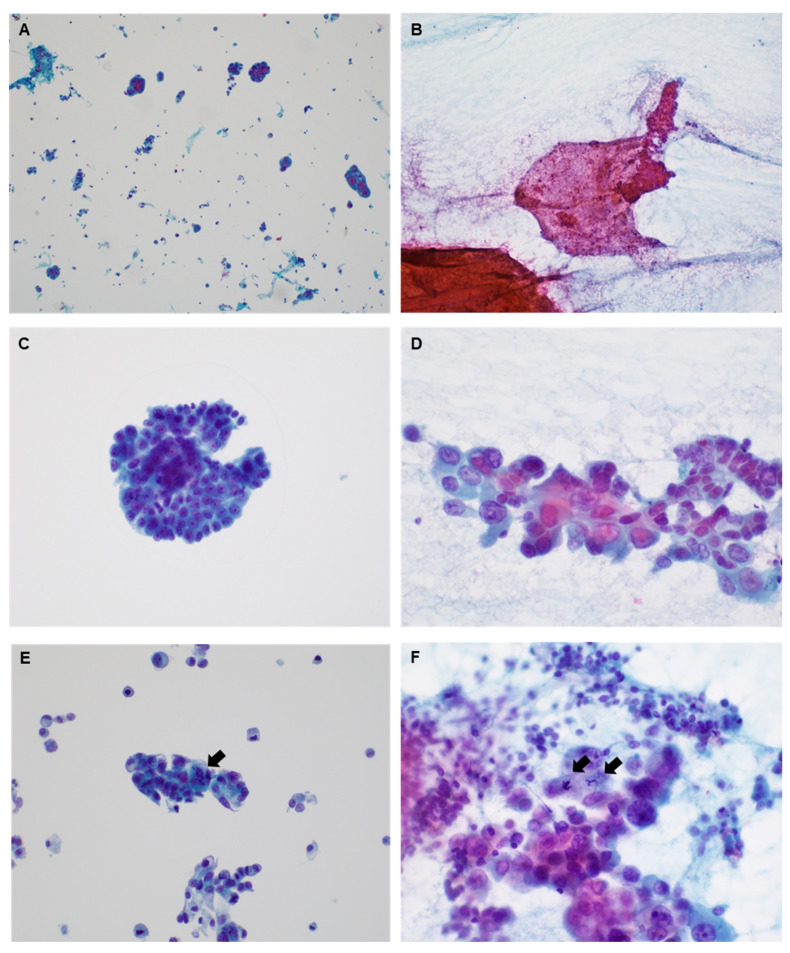
Representative images for endoscopic ultrasonography–fine needle aspiration cytology (EUS-FNAC) with liquid-based preparation (**A**,**C**,**E**) and conventional smear (**B**,**D**,**F**). (**A**,**B**) Low magnification (×100). (**C**–**F**) High magnification (×400; arrow, atypical mitosis).

In the 53 EUS-FNAC cases, cellularity and diagnosis were evaluated by dividing them into LBP and CS. Representative images are shown in Figure 1. Patients with LBP showed 3 insufficient, 22 low, 14 moderate, and 14 high cellularities (Table 2). Cases with CS showed 3 insufficient, 38 low, 10 moderate, and 2 high cellularities. The concordance rate for cellularity was 45.3% (24 of 53 cases). The diagnostic distribution of CS was as follows: Category I (n = 2); Category II (n = 3); Category III (n = 33); Category VI (n = 10); and Category VII (n = 5). The LBP diagnostic distribution was Category I (n = 4), Category II (n = 0), Category III (n = 28), Category VI (n = 7), and Category VII (n = 14). Concordance in cytological diagnoses was observed in 26 of the 53 cases (49.1%) (Table 3).

**Figure 2:**
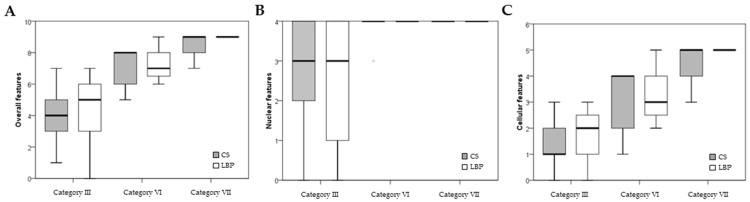
Distributions of cytopathologic features of each diagnostic category in endoscopic ultrasonography–fine needle aspiration cytology (EUS-FNAC) with liquid-based preparation and conventional smear. (**A**) Overall features. (**B**) Nuclear features. (**C**) Cellular features. (CS, conventional smear; LBP, liquid-based preparation).

Cytopathological features based on cytological diagnosis and preparation were evaluated in detail, including the number of features identified out of the 10 cytopathological features. There were no significant differences in the number of cytopathological features between the LBP and CS groups within the same diagnostic category. Gradual increases in the number of cytopathological features were identified between categories III, VI, and VII (Figure 2). However, there was no difference in the nuclear features between categories VI and VII.

### 3.2. Decision Tree Analysis

**Figure 3:**
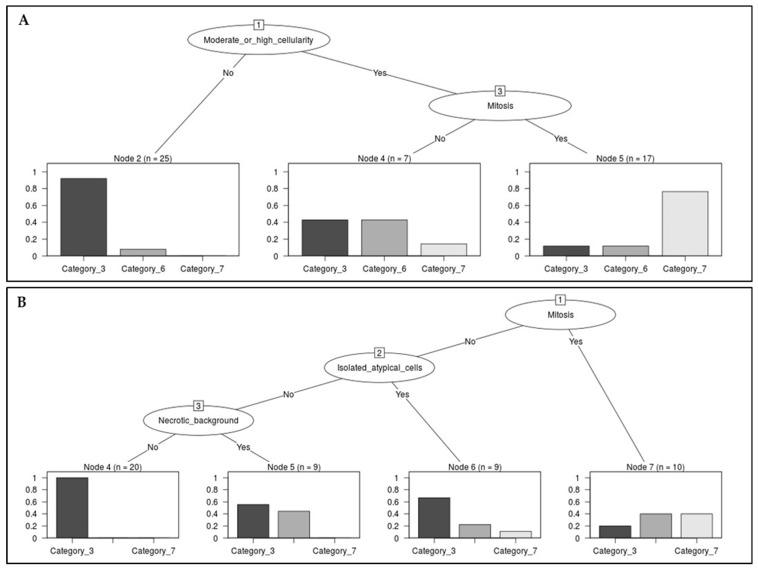
Decision tree for endoscopic ultrasonography–fine needle aspiration cytology (EUS-FNAC) with liquid-based preparation (**A**) and conventional smear (**B**).

Next, we performed decision tree analysis to evaluate the diagnostic impact of the cytopathological features on EUS-FNAC. All patients exhibited considerable nuclear atypia and were diagnosed as atypical, suspected malignancy, or malignant. According to this result, because the impact of nuclear atypia was not significant, a detailed decision tree analysis of additional features was performed by dividing them into LBP and CS groups (Figure 3). Root nodes showed cellularity and mitosis in the LBP and CS groups, respectively (Figure 3). In the assessment of CS, the absence of mitosis was a feature that favored category III. All CS cases with no mitosis, isolated atypical cells, and necrotic background were diagnosed as category III. In the assessment of LBP, if cases with low cellularity showed no mitosis, the cytological diagnosis was category III rather than categories VI or VII (23 of 25 cases with low cellularity; Figure 3A). In LBP with moderate or high cellularity, mitosis favors malignancy.

## 4. Discussion

The comprehensive understanding of cytopathologic features within each category remains elusive in the context of EUS-FNAC for pancreatic lesions. This lack of clarity can lead to diagnostic discord among observers in daily practice. This study aims to enhance the accuracy of EUS-FNAC diagnoses for pancreatic lesions by comprehensively analyzing both similarities and differences in cytopathologic features across categories. It represents the first evaluation of cytologic characteristics and the identification of diagnostic challenges associated with EUS-FNAC in pancreatic lesions using the WHO Reporting System for Pancreaticobiliary Cytopathology. Additionally, a decision tree analysis was employed to compare the cytologic features between LBP and CS.

Cytopathologic examination for pancreatic lesions may encounter limitations due to their anatomical location. In daily practice, diagnosing pancreatic lesions often relies on a restricted sample. Ongoing efforts strive to improve the diagnostic accuracy of EUS-FNAC through the implementation of the PSC and WHO reporting systems [1,19]. Despite sharing many similarities, these reporting systems, as elucidated in previous studies, lack insights into the diagnostic pitfalls encountered in daily practice [1,19]. Moreover, the introduction of ROSE aims to address the challenge of limited samples for diagnosing pancreatic lesions [3]. However, it is important to note that ROSE cannot be applied to the LBP of EUS-FNAC. The comprehensive comparison of cytopathologic features between LBP and CS of EUS-FNAC in the context of pancreatic lesions remains underexplored. Therefore, in daily practice, a thorough comparison of cytopathologic features between LBP and CS in EUS-FNAC becomes imperative. For cases deemed suspected malignancy, repeating an FNA is recommended, especially if preoperative therapy is required. Previous research indicates that category III carries a 30–40% ROM, whereas ROMs escalate to 80–100% for Category VI and 99–100% for Category VII [1]. However, there is limited information regarding potential cytopathologic differences between the LBP and CS of EUS-FNAC with atypical features. Further studies are needed to understand the reasons behind atypical cases not being classified into suspected malignancy or malignancy categories. Additionally, there is a lack of detailed information on the advantages and disadvantages of LBP and CS in determining the diagnosis. While numerous studies have addressed the diagnostic accuracy of LBP and CS in EUS-FNAC, our study specifically aimed to elucidate the cytopathologic features of LBP and CS in pancreatic lesions [3].

A reporting system serves the dual purpose of standardizing terminology across all investigators and minimizing confusion. Moreover, the adoption of a reporting system has the potential to reduce atypical interpretations. The PSC reporting system was introduced in 2014, categorizing findings into six distinct categories, while the WHO reporting system comprises seven categories [19]. Both systems share classifications such as insufficient, benign, atypical, suspected malignant, and malignant [1,19]. Notably, the ROMs for categories labeled as suspected malignancy were 88% and 91% in the PSC and WHO reporting systems, respectively [24]. The ROMs of the atypical category were 50% and 69% in the PSC and WHO reporting systems, respectively. In Hoda’s report, the concordance rate between atypical categories in the PSC and WHO reporting systems was 100%, encompassing 25 cases [20]. However, Hoda et al. reported an ROM of 28% for category III in both the PSC and WHO reporting systems, revealing a significant disparity in ROM counts between reports [20]. This incongruity may stem from unclear diagnostic features within the atypical category. In the WHO reporting system, 12 cytopathologic features were described in the atypical category. Nevertheless, these findings, in isolation, may not provide sufficient clarity to distinguish the atypical category from the suspected malignancy or malignancy categories.

The WHO reporting system outlines the cytopathologic features observed in the atypical category. Moreover, if the cytopathologic features do not align with either benign or malignant characteristics, diagnosing it as an atypical category may be possible. In certain cases, the cytopathologic features of malignancy may be evident, yet the atypical category may be assigned due to low cellularity. However, previous reports have predominantly concentrated on the implications of the atypical category for ROMs [25]. In a systematic review, the ROMs of the “atypical” category ranged from 28% to 100%, highlighting the extensive spectrum within category III and the challenges associated with applying specific criteria for diagnosis [25]. In daily practice, reducing the number of cases diagnosed as atypical category holds significance. The improvement in diagnostic accuracy in category III will necessitate the incorporation of more quantitative diagnostic tools. As previously described, cytopathologic features of malignancy can also manifest in cases labeled as atypical category. Thus, an assessment of the factors influencing the diagnosis of atypical categorization rather than a malignancy becomes essential. To address this, we conducted a study investigating the dependency of the diagnosis on nuclear and cellular features.

The attribution of the atypical category diagnosis was prompted by the presence of a small number of atypical cells, which did not meet the criteria for malignancy or benign [24]. Notably, all cases categorized as III, VI, or VII exhibited nuclear atypia, suggesting that the influence of nuclear features on the diagnosis may be constrained. Consequently, we analyzed other cellular features, excluding nuclear atypia, using a decision tree analysis. Our results contribute to the assessment of the diagnostic utility of cytopathologic features in EUS-FNAC.

The present study evaluated the cellularity of LBP and CS in EUS-FNAC. Insufficient celluarity was identified in three cases each of LBP and CS. Cases with low cellularity accounted for 41.5% and 71.7% of LBP and CS cases, respectively. Notably, our results highlight LBP’s advantage in terms of cellularity compared to CS. The cytologic preparation with CS may exhibit low cellularity or poor fixation, influenced by variations in the investigators’ technique. The application of ROSE enhanced sample adequacy, particularly in cases with low cellularity. In a previous meta-analysis, CS without ROSE demonstrated lower diagnostic accuracy in EUS-FNAC of the pancreas compared to CS with ROSE [3]. However, the impact of ROSE was limited in LBP [3]. In the present study, ROSE was not applied in all cases. Recent reports suggest that a combination of ERCP and EUS may be helpful in the diagnosis of pancreatobiliary lesions [26,27]. However, the effect of the combination on cytologic preparation and yield has not been well reported. We analyzed the data using 53 cases in the present study. Of these, 3 cases were insufficient for diagnosis, which means that 50 cases of CS and 50 cases of LBP were included. In previous reports, both LBP and CS were not performed and compared for cytopathologic features, so despite the small number of cases, we believe the results are significant.

The distribution of cases across categories III, VI, and VII showed no significant difference, with 49 and 48 cases for LBP and CS, respectively. None of the cases fell within category I for either LBP or CS. Category I in each preparation method was complemented by other methods in our study, emphasizing the advantage of evaluating both preparation methods when possible, rather than relying solely on LBP and CS. For the non-diagnostic category I, the ROM ranged from 8 to 57% [24]. The application of ROSE proved helpful in reducing non-diagnostic cases. In cases with a necrotic background, 32 out of 35 were observed in the present study, with 15 and 5 cases in LBP and CS, respectively. Importantly, 12 of the 32 cases with a necrotic background were seen in both LBP and CS. Of the 27 cases with a necrotic background in LBP, three were insufficient and categorized as I, while in CS, only one case fell into category I with a necrotic background. The decision tree analysis highlighted the importance of the necrotic background in CS rather than LBP. The overall concordance rate between LBP and CS was 49.1%, with LBP demonstrating diagnostic superiority in 34.0% of cases compared to 17.0% for CS. This discrepancy may be attributed to an uneven distribution through the same FNA. Among four LBP cases classified as category I, three were diagnosed as category III and one as category VI. In CS cases diagnosed as category I, one was diagnosed as category III and one as category VII.

In the WHO reporting system, atypical mitosis is identified as a hallmark of malignancy. Notably, atypical mitosis was detected in 15 LBP and 4 CS cases, respectively. In this study, we categorized these instances as mitosis, irrespective of atypical mitosis. It is important to highlight that all cases exhibiting atypical mitosis were confined to categories VI and VII. To further assess the importance of atypical mitosis, we conducted a decision tree analysis. Surprisingly, in CS, atypical mitosis did not emerge as a significant factor. Subsequently, we substituted mitosis with atypical mitosis in CS and reran the decision tree analysis, resulting in no notable difference. However, in LBP, a distinct outcome was observed. The root node changed from cellularity to atypical mitosis. This suggests that cases characterized by nuclear atypia and atypical mitosis in LBP can be reliably classified into category VII or VI. Conversely, cases without atypical mitosis, coupled with low cellularity in LBP, predominantly fell into category III.

## 5. Conclusions

This study compared cytopathologic features between the CS and LBP of EUS-FNAC for pancreatic lesions with suspected adenocarcinoma. Furthermore, we evaluated the diagnostic implications of cytopathologic features in both LBP and CS across different diagnostic categories. Notably, cases categorized as III, VI, and VII may present diverse cytopathologic features, such as cellularity, mitosis, and necrotic background, which could pose challenges in accurately diagnosing EUS-FNAC. We were able to identify the significance of each cytopathologic finding in diagnosis using endoscopic ultrasonography–fine needle aspiration cytology in pancreas. The cytopathologic findings that can be applied to daily practice were evaluated and are expected to further increase the value of EUS diagnosis in pancreas. Based on our findings, EUS-FNAC is expected to reduce underdiagnostic or overdiagnostic interpretation in the cytologic diagnosis of pancreatic lesions. Future study with a larger number of cases would be helpful to evaluate the impact of our results on the concordance rate between diagnostic categories.

## Figures and Tables

**Table 1:** Various cytologic features of pancreatic ductal adenocarcinoma in endoscopic ultrasonography–fine needle aspiration cytology.

**Nuclear features**
Irregular nuclear contours (notches, grooves, convolutions)
Nuclear enlargement
Hypochromatic nuclei with parachromatin clearing
Nucleoli
**Additional features**
Isolated atypical cells
Mucinous cytoplasm
Drunken honeycomb architecture
Mitosis, including atypical mitosis
Necrotic background
Moderate or high cellularity

**Table 2:** Concordance in cellularity between conventional smear and liquid-based preparation in endoscopic ultrasonography–fine needle aspiration.

	Conventional Smear				
	Insufficient	Low	Moderate	High	Sum
Liquid-based preparation					
Insufficient	0	2	1	0	3
Low	2	19	1	0	22
Moderate	0	9	4	1	14
High	1	8	4	1	14
Sum	3	38	10	2	53

Numbers in parentheses represent percentage.

**Table 3:** Diagnostic concordance between conventional smear and liquid-based preparation in endoscopic ultrasonography–fine needle aspiration.

WHO Category		Conventional Smear					
		Category I	Category II	Category III	Category VI	Category VI	Sum
Liquid-based preparation	Category I	0	0	3	0	1	4
	Category II	0	0	0	0	0	0
	Category III	1	2	20	4	1	28
	Category VI	0	0	5	2	0	7
	Category VII	1	1	5	3	4	14
	Sum	2	3	33	9	6	53

## Data Availability

The data supporting the findings of this work are available upon reasonable request to the corresponding author.
